# Transcription of the *Sox30* Gene Is Positively Regulated by Dmrt1 in Nile Tilapia

**DOI:** 10.3390/ijms20215487

**Published:** 2019-11-04

**Authors:** Yaohao Tang, Xiaoyan Li, Hesheng Xiao, Minghui Li, Yueqin Li, Deshou Wang, Ling Wei

**Affiliations:** 1 Key Laboratory of Freshwater Fish Reproduction and Development (Ministry of Education), School of Life Sciences, Southwest University, Chongqing 400715, China; tyhwojiushiwo@163.com (Y.T.); 15085043894@163.com (X.L.); xhs162011@email.swu.edu.cn (H.X.); limh@163.com (M.L.); 17723347769@163.com (Y.L.); 2 Key Laboratory of Aquatic Science of Chongqing, School of Life Sciences, Southwest University, Chongqing 400715, China

**Keywords:** Nile tilapia, *Sox30*, transcriptional regulation, Dmrt1

## Abstract

The Sox family member Sox30 is highly expressed in the testis of several vertebrate species and has been shown to play key roles in spermiogenesis. However, its transcription regulation remains unclear. Here, we analyzed the *Sox30* promoter from the teleost fish Nile tilapia (*Oreochromis niloticus*) and predicted a putative cis-regulatory element (CRE) for doublesex and mab-3 related transcription factor 1 (Dmrt1), a male-specific transcription factor involved in male sex differentiation. Transcriptional profiling revealed that *Sox30* and *Dmrt1* similarly exhibited a high expression in tilapia testes from 90 days after hatching (dah) to 300 dah, and the transcription of the *Sox30* gene was reduced about one-fold in the testes of male tilapia with *Dmrt1* knockdown. Further dual-luciferase reporter assay confirmed that *Dmrt1* overexpression significantly promoted transcriptional activity of the *Sox30* promoter and this promotion was decreased following the mutation of putative CRE for Dmrt1 within the *Sox30* promoter. Chromatin immunoprecipitation-based PCR (ChIP-PCR) and electrophoretic mobility shift assay (EMSA) demonstrated that Dmrt1 directly binds to putative CRE within the *Sox30* promoter. These results together indicate that Dmrt1 positively regulates the transcription of the tilapia *Sox30* gene by directly binding to specific CRE within the *Sox30* promoter.

## 1. Introduction

Sox transcription factors, containing a high mobility group (HMG)-box domain for DNA-binding, are common in metazoans and play important roles in various developmental processes, including sex determination, sexual differentiation, gonadal development, neural development [1]. Sox30 is the only member of group H of the Sox family and exists widely among the animal kingdom [2,3,4,5], including human (*Homo sapiens*), mouse (*Mus musculus*), chicken (*Gallus gallus*), Nile tilapia, the channel catfish (*Ictalurus punctatus*), guppy (*Poecilia reticulata*), fathead minnow (*Pimephales promelas*), little skate (*Leucoraja erinacea*), dogfish (*Squalus acanthias*), lancelets (*Branchiotoma belcheri*), and elephant shark (*Callorhinchus milii*). Although the roles of the Sox30 protein in teleost are currently unclear, recent studies in mouse have demonstrated that Sox30 is involved in spermiogenesis and *Sox30* knockout results in abnormality in germ cell development and a complete arrest of spermiogenesis [6,7,8].

Increasing evidence revealed that the *Sox30* gene exhibits a male-biased expression in teleost fish and mouse. In Nile tilapia, *Sox30* is highly expressed in the testis at 90 days after hatching (dah) and 180 dah, especially in the sperms [4,9]. Similarly, mouse Sox30 is predominantly expressed in spermatocytes and round spermatids of the male germ cells during spermiogenesis in testis [7,8]. Previous studies reported that mouse *Sox30* gene is transcriptionally regulated by retinoic acid and by the transcription factor MYBL1 [8,10]. However, the regulatory mechanism underlying the male-biased expression of the *Sox30* gene in animals remains largely unknown.

Doublesex and mab-3 related transcription factor 1 (Dmrt1) contains a zinc finger-like DM domain for DNA binding and is widely considered to be involved in male sex determination, differentiation and maintenance in vertebrates [11,12,13]. In teleosts, such as zebrafish and Nile tilapia, *Dmrt1* is dominantly expressed in the testis [14,15]. The loss of function of zebrafish *Dmrt1* results in a lack of germ cells in males and sex reversal from male to female [16]. Our previous studies have demonstrated that *Dmrt1* knockdown in Nile tilapia causes testis degradation, and *Dmrt1* overexpression in XX fish results in sex reversal [17,18]. In addition, Dmrt1 is a bifunctional transcriptional regulator [19]. For example, in Nile tilapia, our lab has found that Dmrt1 can not only activate the transcription of the gonadal soma-derived factor (*Gsdf*) gene critical for testicular differentiation [20] but also inhibit the expression of the *Cyp19a1a* gene, a gene encoding aromatase responsible for the biosynthesis of estrogens from androgens [21]. Therefore, the similarities in both expression profiles and functions between *Sox30* and *Dmrt1* raise a possibility that Dmrt1 may be also involved in regulating male-biased expression of the *Sox30* gene in teleost and other animals.

The Nile tilapia as a vertebrate species is a good model for the study of sex determination and differentiation, due to the availability of genome sequences and transcriptome data [22,23], the breeding of monosex fish [24], the establishment of CRISPR/Cas9 editing technology [18], and economical values of being aquatic fish. Given that the *Sox30* gene is highly expressed in Nile tilapia testis, here, we investigated the regulatory function of Dmrt1 on *Sox30* transcription in Nile tilapia. We observed that both *Sox30* and *Dmrt1* are highly co-expressed in XY Nile tilapia gonads (testis) and that *Sox30* is less transcribed in the testis of Nile tilapia with *Dmrt1* knockdown (heterozygous mutant). We also predicted a putative cis-regulatory element (CRE) for Dmrt1 within the sequence of the Nile tilapia *Sox30* promoter. Further experiments confirmed that Dmrt1 directly binds to that putative CRE within the *Sox30* promoter to activate its transcriptional activity. Altogether, our results demonstrate that Dmrt1 directly and positively regulated the transcription of the male-biased *Sox30* gene in Nile tilapia testis.

## 2. Results

### 2.1. Proximal Promoter of the Nile Tilapia Sox30 Gene Contains a Putative CRE for Dmrt1

**Figure 1:**
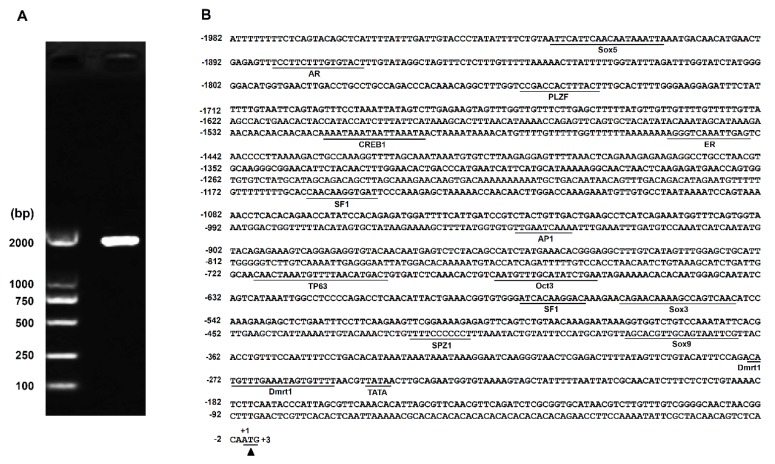
Cloning and sequencing of the Nile tilapia *Sox30* promoter. (**A**) Cloning of the proximal promoter region containing a 1982 bp sequence upstream of the translational initiation site (ATG) of the Nile tilapia *Sox30* gene. The first nucleotide of the transcription initiation site (ATG) is defined as +1 and its upstream region is indicated as minus number. (**B**) Bioinformatics prediction of potential cis-regulatory elements (CREs) for transcription factors within proximal promoter of the Nile tilapia *Sox30* gene. The predicted TATA box and CREs for the binding of various transcription factors are underlined. The putative translation start site is indicated by the solid triangle. The potential core binding site for doublesex and mab-3 related transcription factor 1 (Dmrt1) located in the region from −274 to −254 within the Nile tilapia *Sox30* promoter.

Given that transcription factor Dmrt1 is a master regulator in male sex determination and differentiation in teleost and mouse [12,13,17], and *Sox30* is predominantly expressed in male gonads (testis) in teleost and mouse [4,9,14], we questioned whether the Dmrt1 might be involved in transcriptional regulation of the *Sox30* gene. Therefore, we cloned the proximal promoter containing 1982 bp sequence upstream of the translational start site of the tilapia *Sox30* gene (Figure 1A). MatInspector-based in slico predication identified that, in addition to several transcription factors (SF1, Sox5, androgen receptor-AR, and estrogen receptor-ER), there is a putative CRE involved in Dmrt1 DNA binding located within the Nile tilapia *Sox30* promoter (Figure 1B). This data suggests that Dmrt1 may directly bind to the promoter of the Nile tilapia *Sox30* gene to regulate its transcription. Interestingly, we analyzed the promoter sequences of the *Sox30* genes from other organisms and also found putative CREs for Dmrt1 within these promoters (supplementary Figure S1). This suggests that Dmrt1 may be conservatively involved in the regulation of the *Sox30* transcription in animals.

### 2.2. Sox30 and Dmrt1 Are Prominently Expressed in Male Nile Tilapia Gonads

**Figure 2:**
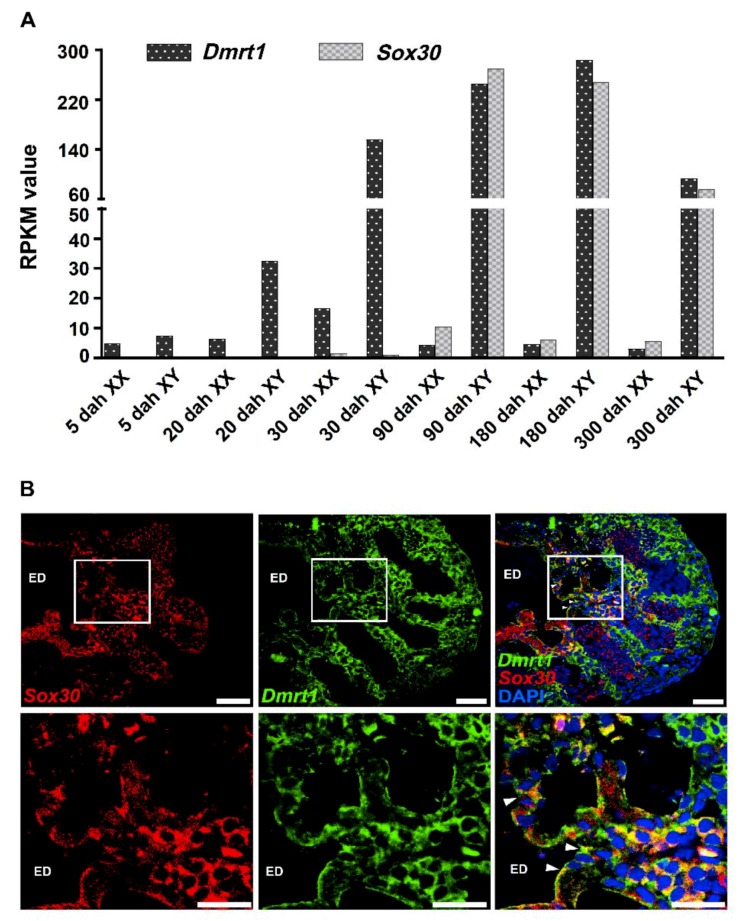
Expression patterns of *Sox30* and *Dmrt1* in the Nile tilapia testis. (**A**) Transcriptome-based analysis of expression patterns of *Sox30* and *Dmrt1* in the gonads of the Nile tilapia during different developmental stages. The Reads Per Kilobase per Million mapped reads (RPKM) value was used to evaluate the expression level. dah, days after hatching; XX, female gonad; XY, male gonad. (**B**) Fluorescent in situ hybridization analysis of expression patterns of *Sox30* and *Dmrt1* in the gonads of male Nile tilapia at 90 dah. Double-colored fluorescent in situ hybridization was carried out on cross sections of male gonads at 90 dah. The below images were separately enlarged according to the regions indicated above images. The result shows that *Sox30* and *Dmrt1* are co-expressed in the epithelial cells comprising the efferent duct. White arrowheads indicate epithelial cells. scale bars =20 μm. ED, efferent duct.

To evaluate the possible regulation of Dmrt1 on the transcription of the Nile tilapia *Sox30* gene, we further profiled the temporal expressions of *Sox30* and *Dmrt1* in gonads during Nile tilapia development. Transcriptome-based analysis revealed that *Sox30* exhibited male-biased expression in the gonads, being prominently expressed in XY gonad (testis) from 90 dah to 300 dah (Figure 2A). Similarly, the *Dmrt1* gene was expressed in the XY gonad during Nile tilapia development, from 5 dah to 300 dah, especially after 30 dah (Figure 2A). Furthermore, we conducted in situ hybridization experiments to determine subcellular localization of the expression of both *Dmrt1* and *Sox30* in Nile tilapia testis at 90 dah. Previous study in Nile tilapia has shown that *Dmrt1* is expressed in the Sertoli and epithelial cells of the intratesticular efferent duct at 70 dah and 100 dah [14]. Our results revealed that *Dmrt1* and *Sox30* were co-expressed in epithelial cells of the intratesticular efferent duct in the testis of Nile tilapia at 90 dah (Figure 2B).

### 2.3. Sox30 Transcription Was Decreased in the Testis of Nile Tilapia with Dmrt1 Knockdown

**Figure 3:**
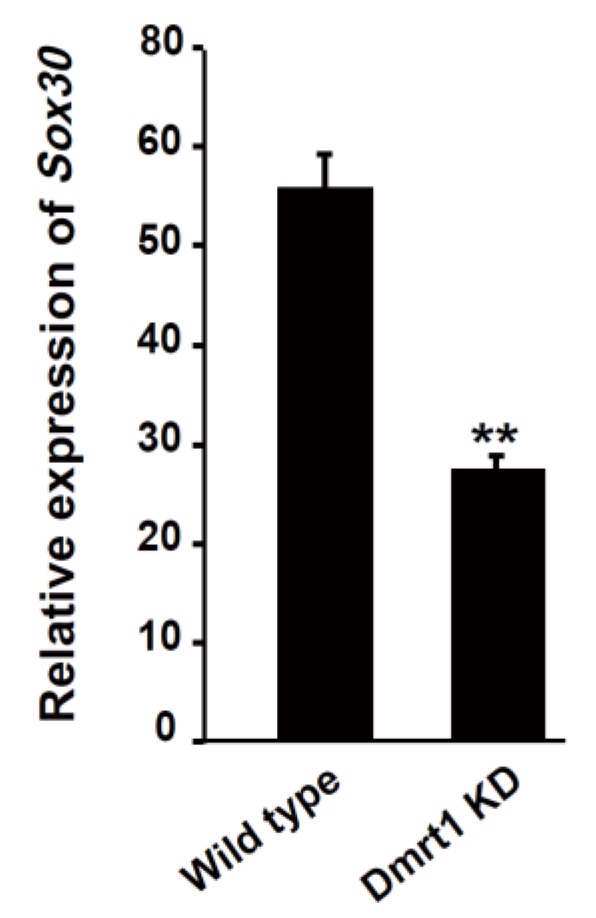
Change of *Sox30* expression in the testes of *Dmrt1*-deficient Nile tilapia. *Sox30* expression was detected by quantitative RT-PCR in the testes of male Nile tilapia with *Dmrt1* knockdown at 90 dah. KD, knockdown. The experiments were independently repeated three times. The data are presented as the means ± SE of the triplicates. **, *p* < 0.01, compared with the control.

We previously reported that *Dmrt1* knockdown mediated by transcription activator-like effector nucleases (TALENs) or CRISPR/Cas9 in tilapia resulted in testicular regression and an increase in the expression of its target gene *Cyp19a1a* in testis [17,18]. Because both *Dmrt1* and *Sox30* displayed a high expression in the XY gonad (testis) of Nile tilapia at 90 dah (Figure 2A), we further investigated the effects of *Dmrt1* knockdown on the expression of the *Sox30* gene in the testis of the Nile tilapia at 90 dah. Notably, quantitative RT-PCR analysis showed that compared with wild type of Nile tilapia, *Sox30* transcription was significantly decreased in the testes of *Dmrt1*-deficient Nile tilapia (Figure 3). This result indicates that Dmrt1 may positively regulate the transcription of the *Sox30* gene in Nile tilapia testis.

### 2.4. The Nile Tilapia Sox30 Promoter Is Sensitive to Dmrt1

**Figure 4:**
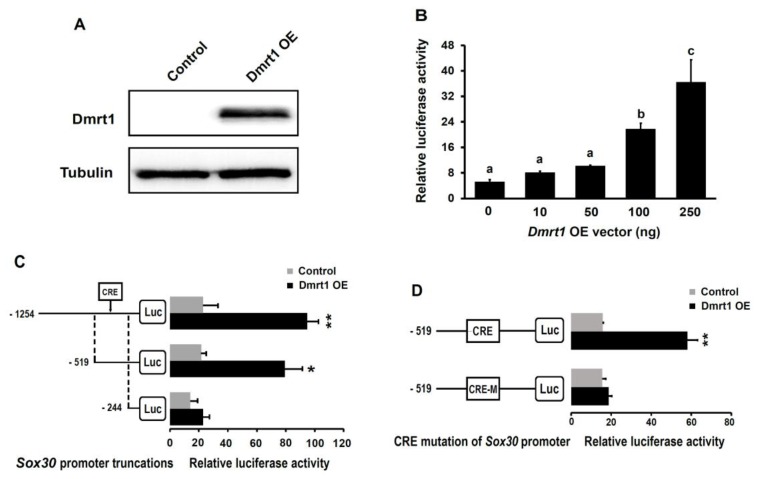
Effects of *Dmrt1* overexpression on the activity of the Nile tilapia *Sox30* promoter. (**A**) Western blotting validation of the overexpression of the Nile tilapia *Dmrt1* gene in human HEK293 cells. Cell lysates and total proteins were prepared separately from the cells with and without *Dmrt1* overexpression. Anti-Dmrt1 antibody and anti-Tubulin antibody were used. (**B**) Dosage-dependent effects of *Dmrt1* overexpression on the luciferase expression driven by the *Sox30* promoter in human HEK293 cells. Different dosage of pcDNA3.1-*Dmrt1* overexpressing vectors were separately co-transfected into HEK293 cells with a construct containing a luciferase reporter driven by the Nile tilapia *Sox30* promoter. An empty construct without *Dmrt1* was used as the control. Luciferase reporter analysis indicates that *Dmrt1* overexpression promoted the activities of the Nile tilapia *Sox30* promoter in a dosage-dependent manner. (**C**) Effects of *Dmrt1* overexpression on the luciferase expression driven by several 5′-truncated regions of the *Sox30* promoter in HEK293 cells. (**D**) Effects of *Dmrt1* overexpression on the activity of the Nile tilapia *Sox30* promoter with a mutation of putative CRE for Dmrt1. The data represent the means + SE (*n* = 3). Different letters above the error bars in (**B**) and (**C**) indicate statistical differences at *p* < 0.05, as determined by one-way ANOVA followed by the post hoc test in (**B**). *, *p* < 0.05; and **, *p* < 0.01 compared with the control in (**C**) and (**D**). OE, overexpression. Luc, luciferase; M, mutation.

We performed dual-luciferase reporter assays in human HEK293 cells to evaluate the effects of Dmrt1 on the activity of the Nile tilapia *Sox30* promoter. We constructed the pcDNA3.1-*Dmrt1* vector for overexpressing the Nile tilapia *Dmrt1*. Western blot analysis revealed that the Nile tilapia Dmrt1 protein could be expressed in HEK293 cells with transient transfection of pcDNA3.1-*Dmrt1* vector (Figure 4A). Simultaneously, a construct containing a luciferase reporter gene under the control of the proximal promoter of the Nile tilapia *Sox30* gene was generated and then co-transfected with different dosage of the pcDNA3.1-*Dmrt1* vectors into HEK293 cells. Subsequent analysis showed that Dmrt1 overexpression significantly promoted the activities of the Nile tilapia *Sox30* promoter in a dosage-dependent manner (Figure 4B).

To examine the roles of the predicted CRE for the Dmrt1 protein within the Nile tilapia *Sox30* promoter, we carried out a truncation analysis for the Nile tilapia *Sox30* promoter. Three luciferase reporter constructs driven by 5′-flanking serially truncated fragments of the Nile tilapia *Sox30* promoter were generated and separately co-transfected with the pcDNA3.1-*Dmrt1* vectors into HEK293 cells. Subsequent analysis showed that compared with the control, *Dmrt1* overexpression enhanced the transcriptional activity of the truncated *Sox30* promoter containing a putative CRE for Dmrt1, reaching to about four-folds (Figure 4C). But, the truncated *Sox30* promoter lacking putative CRE for Dmrt1 did not respond to *Dmrt1* overexpression (Figure 4C). Similarly, the tilapia *Sox30* promoter obviously lost the *Dmrt1* overexpression-induced upregulation of its transcriptional activity following the mutation of putative CRE for Dmrt1 (Figure 4D). Altogether, these results strongly support that Dmrt1 positively regulates the transcription of the Nile tilapia *Sox30* gene via a specific CRE for Dmrt1 within its promoter.

### 2.5. Dmrt1 Directly Binds to a Specific CRE Within the Nile Tilapia Sox30 Promoter

**Figure 5:**
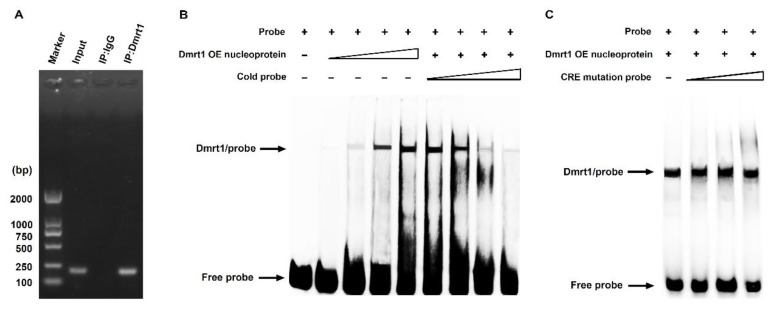
Direct binding of Dmrt1 to specific CRE within the Nile tilapia *Sox30* promoter. (**A**) Chromatin immunoprecipitation (ChIP) assays of the binding of Dmrt1 to putative CRE in the *Sox30* promoter in the testis of Nile tilapia at 90 dah. A primer pair covering the *Sox30* promoter region containing putative CRE for Dmrt1 was used for PCR examination in the products precipitated from the Nile tilapia testes with an anti-Dmrt1 antibody. (**B**) Electrophoretic mobility shift assay (EMSA) analysis of the binding of Dmrt1 to specific CRE in the Nile tilapia *Sox30* promoter. Specific probe against putative CRE for Dmrt1 was designed. The unlabeled intact probes could compete for the binding of Dmrt1 to the labeled intact probes in a dose-dependent manner. (**C**) The unlabeled probe with a mutation of putative CRE for Dmrt1 could not suppress the binding of Dmrt1 to the labeled intact probe.

We finally investigated whether Dmrt1 could directly bind to the specific CRE for Dmrt1 within the Nile tilapia *Sox30* promoter. First, a chromatin immunoprecipitation (ChIP) analysis revealed that based on a PCR amplification with a specific primer pair covering the DNA region containing putative CRE for Dmrt1, a positive DNA fragment was detectable in the products precipitated with the antibody against Dmrt1 in Nile tilapia testes at 90 dah, as demonstrated by its size similar to the PCR products using input DNA as a template (Figure 5A). Notably, this DNA fragment could not be detected in the products precipitated with the nonspecific IgG as a negative control (Figure 5A).

We further performed an electrophoretic mobility shift assay (EMSA) to determine the direct binding of Dmrt1 to putative CRE within the Nile tilapia *Sox30* promoter. The nucleoproteins were isolated from HEK293 cells overexpressing the Nile tilapia *Dmrt1* gene and the oligonucleotide probes covering putative CRE for Dmrt1 in the Nile tilapia *Sox30* promoter were synthesized. The EMSA results revealed that the nucleoproteins containing Dmrt1 protein could directly bind to the 5′-Biotin-labeled probes in a dose-dependent manner, and this binding could be competitively impaired by the unlabeled cold probes (Figure 5B). However, the unlabeled probes with a mutation of putative CRE for Dmrt1 had no competence (Figure 5C). These results, together with ChIP data, indicate that Dmrt1 can directly bind to specific CRE within the Nile tilapia *Sox30* promoter.

## 3. Discussion

The DM transcription factor Dmrt1 has been well-characterized as a master regulator in male sex determination, differentiation, and maintenance in vertebrates [12,13,25,26,27]. The knockdown of the tilapia *Dmrt1* gene leads to an abnormality in male sexual development, especially testicular regression in testis [17,18]. Increasing evidence has demonstrated that Dmrt1 is predominantly expressed in testis and plays a transcriptional regulation in both the activation of male-biased genes, like *Sohlh1*, *Gsdf*, and *Sox9b* [3,20,28], and the repression of the transcription of female genes, like *Foxl2* and *Cyp19a1a* [18,21,29]. Our results together with previous evidence in the teleost fish Nile tilapia demonstrate that *Sox30 and Dmrt1* exhibit a co-expression in testis and Dmrt1 positively regulates the transcription of the *Sox30* gene via a direct binding to a specific CRE within its promoter.

Sox30 is a member of the Sox transcription factor family and exists in many animals, including teleosts and mammals [2,9,30]. It has been demonstrated in mouse that the *Sox30* gene is predominantly expressed in testis and *Sox30* knockout results in an abnormal spermiogenesis [7,8]. In the present study, we observed in Nile tilapia that the *Sox30* gene is expressed in epithelial cells of the intratesticular efferent duct of the testis. The present finding, together with previous observation [14], further confirmed that *Dmrt1* expression is co-located with *Sox30* expression in testis efferent duct. These results suggested that *Sox30* and *Dmrt1* may be involved in regulating the development of testis efferent duct. As is well known, the efferent duct is responsible for the absorption of fluid from the lumen, spermatozoon maturation, spermiophagy, and secretion of steroid hormone [31,32,33]. Recent studies reported that the male sterility caused by the mutation of either Nile tilapia *Cyp19a1b* or mouse *Gemc1* is primarily due to the defect of efferent duct development [34,35]. Thus, further work will be needed to clarify the roles of both *Sox30* and *Dmrt1* in the efferent duct of the Nile tilapia testis.

A striking finding in this study is that Dmrt1 positively regulates the transcription of the *Sox30* gene in Nile tilapia. This conclusion is supported by the following evidences. First, the transcription of the *Sox30* gene in Nile tilapia testis is obviously attenuated by *Dmrt1* knockdown. Second, the overexpression of the Nile tilapia *Dmrt1* gene enhanced transcriptional activities of the *Sox30* promoter in which a specific CRE for the binding of Dmrt1 is included. Third, ChIP-PCR and EMSA assay demonstrated that Dmrt1 could directly bind to specific CRE within the Nile tilapia *Sox30* promoter. This finding helps to better understand the regulatory network involving in male sex determination and differentiation, as well as male gland (testis) development.

## 4. Materials and Methods

### 4.1. Animals and Cell Line

Nile tilapia fishes were reared in recirculating freshwater tanks at 26 °C before use. Animal experiments were conducted in accordance with the regulations of the Guide for Care and approved by the Institutional Animal Care and Use Committee of Southwest University (No. IACUC-20181015-12, 15 October 2018). Moreover, the human embryonic kidney-derived HEK293 cell line were maintained in Dulbecco’s Modified Eagle’s medium (DMEM) containing 10% heat inactivated fetal bovine serum, supplemented with penicillin and streptomycin at 37 °C in a humidified atmosphere containing 5% CO_2_.

### 4.2. Quantitative RT-PCR

Total RNA (2.0 µg) was extracted from the testes of wild type and *Dmrt1* mutant Nile tilapia fishes at 90 dah and subsequently was treated with DNase I to eliminate the genomic DNA contamination. The cDNA for RT-PCR examination was synthesized by using the PrimeScript RT Master Mix Perfect Real Time kit (Takara, Japan). Quantitative RT-PCR analysis of the *Sox30* mRNA expression level in tilapia testis was conducted according to the instructions of the SYBR1 Premix Ex TaqTM II kit (Takara, China). The relative abundance was evaluated using the following formula as described previously [36]: R = 2^−ΔΔ*C*t^. The beta-actin gene was used as a reference gene. The experiments were independently repeated three times. Primers used for RT-PCR are listed in Supplemental Table S1.

### 4.3. Fluorescent In Situ Hybridization

Double-colored fluorescent in situ hybridization-based expression profiling was performed as described as previously [3]. Briefly, the cDNA fragments of tilapia *Dmrt1* (including 301 bp coding sequence and 207 bp 3′ untranslated region) and *Sox30* (including 287 bp coding sequence and 168 bp 3′ untranslated region) were amplified and cloned into pGEM-T Easy vector, respectively. The related primers are listed in supplementary Table S1. The linearized pGEM-T Easy-*Dmrt1* and pGEM-T Easy-*Sox30* plasmids were used as the templates for probe preparation. The probes against *Dmrt1* and *Sox30* were labeled with digoxigenin (DIG) and fluorescein by using RNA labeling kits (Roche, Germany), respectively. In addition, the gonads from male tilapia at 90 dah were fixed in 4% paraformaldehyde in 0.1 mol/L phosphate buffer pH 7.4 at 4 °C overnight. The samples were embedded in paraffin and cross-cut into 5 µm sections. The sections were hybridized with DIG-labeled *Dmrt1* and fluorescein-labeled *Sox30* RNA probes at 60 °C overnight. The first signal was detected by horseradish peroxidase (HRP)-conjugated anti-DIG antibody (Roche, Germany) with the TSA^TM^ Plus Tetramethylrhodamine (TMR) system (PerkinElmer, Boston, MA, USA) according to the manufacturer’s instructions. In order to detect the second signal, the sections were incubated in 3% H_2_O_2_ for 60 min to deactivate the HRP from the first staining. HRP-conjugated anti-fluorescein antibody (Roche, Germany) was then added to the sections, followed by detection with the TSA^TM^ Plus Fluorescein system. Finally, the sections were stained with 4′,6′-diamidine-2-phenylindole- dihydrochloride (DAPI) (Invitrogen, Carlsbad, CA, USA). Confocal images were collected on a Zeiss LSM 880 Laser Scanning Microscope.

### 4.4. Promoter Cloning and Bioinformatics Analysis

The proximal promoter region of the Nile tilapia *Sox30* gene, from −1982 bp to −1 bp relative to the translational start site, was obtained by a PCR reaction with Nile tilapia genomic DNA as a template. Then, the PCR product was cloned into the pGL3-Basic Vector for sequencing. The related primers are listed in supplementary Table S1. Potential CREs for different transcription factors within the tilapia *Sox30* promoter were predicted by using the programs of MatInspector (http://www.genomatix.de) or AnimalTFDB2.0 (http://bioinfo.life.hust.edu.cn/AnimalTFDB/#!/).

### 4.5. Dual-Luciferase Reporter Assay

Dmrt1 regulation on the activity of the Nile tilapia *Sox30* promoter was determined based on dual-luciferase reporter assay as described previously [3,37]. Briefly, the pcDNA3.1-*Dmrt1* overexpression vector for overexpressing the Nile tilapia *Dmrt1* gene in HEK293 cells and a series of constructs containing a luciferase reporter gene under the control of either different 5′-truncations of the *Sox30* promoter or the *Sox30* promoter with a mutation of putative CRE for Dmrt1 were separately generated. Site-directed mutagenesis was performed by using the MutanBEST Kit (TaKaRa, Dalian, China). The related primers are listed in supplementary Table S1. Total proteins were prepared from HEK293 cells with transient transfection of pcDNA3.1-*Dmrt1* vector to check *Dmrt1* overexpression by western blotting analysis. The following antibodies were used, including rabbit anti-Dmrt1 serum (1:500, prepared by Abiotech, Jinan, China), mouse anti-alpha tubulin (1:1000; Beyotime, Shanghai, China), mouse anti-HRP (1:1000, Beyotime, Shanghai, China), and rabbit anti-HRP (1:1000, Beyotime, Shanghai, China). Subsequently, the pcDNA3.1-*Dmrt1* overexpression vector was separately co-transfected into HEK293 cells with each of the constructs containing a luciferase reporter gene driven by different varieties of the Nile tilapia *Sox30* promoter. At 48 h after transfection, we harvested the cells and measured the relative luciferase activity by normalizing the firefly luciferase level to the Renilla luciferase level according to the protocol for the dual luciferase assay system (Promega, Madison, WI, USA).

### 4.6. Electrophoretic Mobility Shift Assay (EMSA)

To test the binding of Dmrt1 to potential CRE in the Nile tilapia *Sox30* promoter, EMSA experiments were performed as described previously [3,38]. Briefly, according to the manufacturer’s protocol for the Chemiluminescent EMSA Kit (Beyotime, Shanghai, China), biotin-labeled and unlabeled double-stranded DNA probes containing intact CRE for Dmrt1 were prepared. The probe with a mutation of the Dmrt1 CRE was also prepared. The related primers are listed in supplementary Table S1. Nuclear proteins were extracted from HEK293 cells overexpressing the Nile tilapia *Dmrt1* gene with NE-PER Nuclear and Cytoplasmic Extraction Reagents (Thermo Scientific, Waltham, MA, USA). Then, 3–5 μg nuclear protein was incubated with 500 nM biotin-labeled probes in 1× binding buffer for 10 min at room temperature. For the competition analysis, a 50-fold molar excess of the unlabeled or mutated probe was added to the nuclear extracts prior to the addition of the labeled probe. Protein-DNA complexes were separated on 4% polyacrylamide gels in 0.5× TBE via electrophoresis at 100 V for 90 min and transferred to nylon membranes. The biotin-labeled DNA on the membrane was detected by using the chemiluminescent EMSA kit.

### 4.7. Chromatin Immunoprecipitation (ChIP)

We further conducted ChIP assays to confirm the direct binding of Dmrt1 to its CRE within the Nile tilapia *Sox30* promoter according to the manufacturer’s instructions (Millipore, Billerica, MA, USA). Briefly, the testes from Nile tilapia at 90 dah were fixed with 37% formaldehyde to cross-link chromatin. The cross-linked chromatins were then sonicated to shear into fragments of 100–500 bp in length. Immunoprecipitation assays were performed using anti-Dmrt1 and nonspecific rabbit IgG antibodies. The purified DNA from the immunoprecipitated chromatin was used as a template for PCR amplification. The primers used to amplify the specific region covering putative CRE for Dmrt1 were listed in Table S1. The PCR products were electrophoresed in 2% agarose gels.

### 4.8. Statistical Analysis

Data were expressed as the mean ± SE of three independent biological replicates. The statistical significance of differences between data means was determined using a two-tailed Student’s t-test or One-way ANOVA, followed by Turkey test for multiple comparisons performed using SPSS 22 software. *p* < 0.05 was statistically significant; *p < 0.05, **p < 0.01, and ***p < 0.001.

## Supplementary Materials

Supplementary materials can be found at https://www.mdpi.com/1422-0067/20/21/5487/s1.

## Abbreviations

HMG	high mobility group
Dah	days after hatching
CRE	cis-regulatory element
EMSA	electrophoretic mobility shift assay
ChIP	chromatin Immunoprecipitation
KD	knockdown
OE	overexpression
