# Supplementary material for: Transcription of the Sox30 Gene Is Positively Regulated by Dmrt1 in Nile Tilapia
Source: Int J Mol Sci. 2019 Nov 4;20(21):5487. doi: 10.3390/ijms20215487 (PMC6862701; doi:10.3390/ijms20215487)
Supplement: Supplementary file 1 [file ijms-20-05487-s001.zip › Supplementary files/Supplementary Table S1.docx]

**Supplementary Table S1 Primers used in this study**

| **Purpose** | **Primer name** | **Primer sequence (5**' **to 3**'**)** |
| --- | --- | --- |
| Quantitative RT-PCR | *Sox30*-F | GATTGTCCAGTCAGAAAAAAGGGTC |
|  | *Sox30*-R | GCGAGGTTTGGGTGAAGTGGTC |
|  | *β-Actin*-F | GGCATCACACCTTCTACAACGA |
|  | *β-Actin*-R | ACGCTCTGTCAGGATCTTCA |
| Promoter cloning | *Sox30*-1982-F | TTTATTTTTTCTCAGTACAGCTCAT |
|  | *Sox30*-1982-R | TGTGAGACTGTTGTAGCGAATAT |
|  | *Sox30*-1254-F | AAATGTTGTGCCTAATAAAATCC |
|  | *Sox30*-1254-R | TTTTGGAAGGTTCTGTGTGTGTG |
|  | *Sox30*-519-F | CAAGAAGTTCGGAAAAGAGAGTT |
|  | *Sox30*-519-R | TTTTGGAAGGTTCTGTGTGTGTG |
|  | *Sox30*-244-F | CTTGCAGAATGGTGTAAAAGTAGC |
|  | *Sox30*-244-R | TTTTGGAAGGTTCTGTGTGTGTG |
|  | *Sox30*-519-M-F | CCCCCCCCCCCCCCACGTTATAACTTGCAGAATGG |
|  | *Sox30*-519-M-R | AAACATGTCTGGAAATGTACAG |
| ChIP PCR | *Sox30*-CRE-F | TTCCAATTTTCCTGACACATAAA |
|  | *Sox30*-CRE-R | CAGAGAGAAAGATGTTGCGATAA |
| EMSA | Biotin-*Sox30*-CRE-F | TTCTGTACATTTCCAGACATGTTTGAAATAGTGTTTTAACGTTATAACTT |
|  | *Sox30*-CRE-F | TTCTGTACATTTCCAGACATGTTTGAAATAGTGTTTTAACGTTATAACTT |
|  | *Sox30*-CRE-R | AAGTTATAACGTTAAAACACTATTTCAAACATGTCTGGAAATGTACAGAA |
|  | *Sox30*-CRE-M-F | TTCTGTACATTTCCAGACATGTTTCCCCCCCCCCCCCCACGTTATAACTT |
|  | *Sox30*-CRE-M-R | AAGTTATAACGTGGGGGGGGGGGGGGAAACATGTCTGGAAATGTACAGAA |
| ISH | *Dmrt1*-probe -F | CAGCGTGTCGTCTCAGTACCG |
|  | *Dmrt1*-probe -R | CCTGAACTGGATTTAAGAGCTAAAA |
|  | *Sox30*-probe -F | CTGTCCCCTATGTGTCCAGTCTAAG |
|  | *Sox30*-probe -R | CATCAGTAGCAATGAAAAAGCACAA |

Note: F indicates forward primer and R indicates reverse primer.
