# Supplementary figures and images for: Transcription of the Sox30 Gene Is Positively Regulated by Dmrt1 in Nile Tilapia
Source: Int J Mol Sci. 2019 Nov 4;20(21):5487. doi: 10.3390/ijms20215487 (PMC6862701; doi:10.3390/ijms20215487)

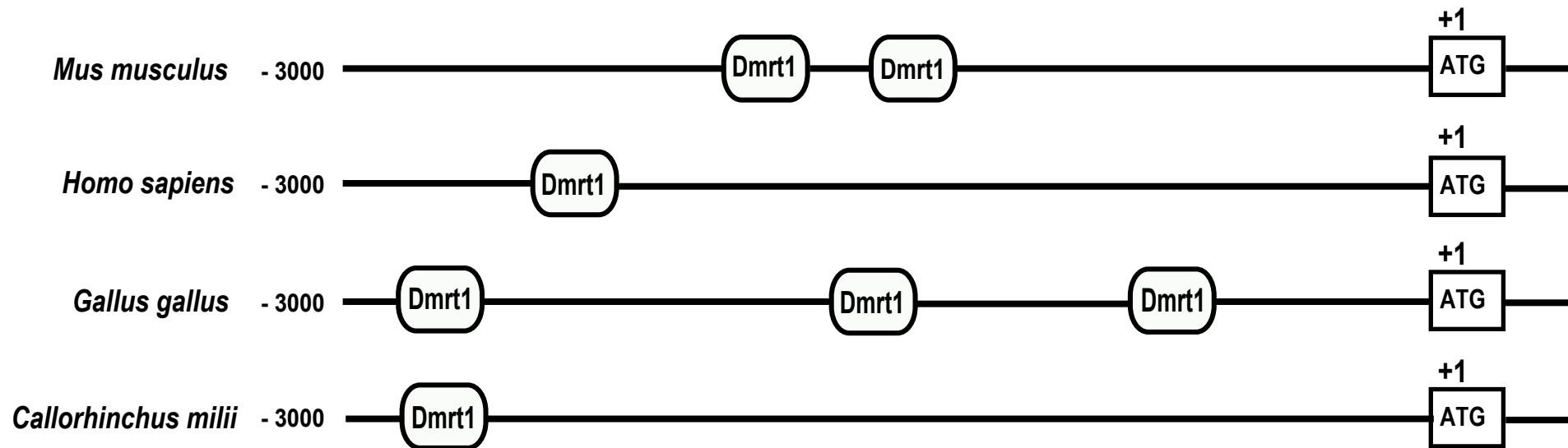

Supplement: Supplementary file 1 [file ijms-20-05487-s001.zip › Supplementary files/Supplementary Figure S1.pdf]
